# Suppression by central adenosine A3 receptors of the cholinergic defense against cardiovascular aberrations of sepsis: role of PI3K/MAPKs/NFκB signaling

**DOI:** 10.3389/fphar.2024.1418981

**Published:** 2024-06-20

**Authors:** Amany E. El-Naggar, Mai M. Helmy, Sahar M. El-Gowilly, Mahmoud M. El-Mas

**Affiliations:** ^1^ Department of Pharmacology and Toxicology, Faculty of Pharmacy, Alexandria University, Alexandria, Egypt; ^2^ Department of Pharmacology and Toxicology, College of Medicine, Kuwait University, Kuwait City, Kuwait

**Keywords:** sepsis, nicotine, adenosine A3 receptors, mitogen activate protein kinase, tumor necrosis factor alpha

## Abstract

**Introduction:** Despite the established role of peripheral adenosine receptors in sepsis-induced organ dysfunction, little or no data is available on the interaction of central adenosine receptors with sepsis. The current study tested the hypothesis that central adenosine A3 receptors (A3ARs) modulate the cardiovascular aberrations and neuroinflammation triggered by sepsis and their counteraction by the cholinergic antiinflammatory pathway.

**Methods:** Sepsis was induced by cecal ligation and puncture (CLP) in rats pre-instrumented with femoral and intracisternal (i.c.) catheters for hemodynamic monitoring and central drug administration, respectively.

**Results:** The CLP-induced hypotension, reduction in overall heart rate variability (HRV) and sympathovagal imbalance towards parasympathetic predominance were abolished by i.v. nicotine (100 μg/kg) or i.c. VUF5574 (A3AR antagonist, 2 µg/rat). In addition, the selective A3AR agonist, 3-iodobenzyl-5′-N-methylcarboxamidoadenosine IB-MECA, 4 µg/rat, i.c.) exaggerated the hypotension and cardiac autonomic dysfunction induced by sepsis and opposed the favorable nicotine actions against these septic manifestations. Immunohistochemically, IB-MECA abolished the nicotine-mediated downregulation of NFκB and NOX2 expression in rostral ventrolateral medullary areas (RVLM) of brainstem of septic rats. The inhibitory actions of IB-MECA on nicotine responses disappeared after i.c. administration of PD98059 (MAPK-ERK inhibitor), SP600125 (MAPK-JNK inhibitor) or wortmannin (PI3K inhibitor). Moreover, infliximab (TNFα inhibitor) eliminated the IB-MECA-induced rises in RVLM-NFκB expression and falls in HRV, but not blood pressure.

**Conclusion:** Central PI3K/MAPKs pathway mediates the A3AR counteraction of cholinergic defenses against cardiovascular and neuroinflammatory aberrations in sepsis.

## 1 Introduction

Sepsis is a life-threatening inflammatory condition that results in continuous activation of inflammatory and coagulation cascades and end-organ damage (Gyawali et al., 2019). Among the various signaling pathways involved in the hyperinflammatory phase of sepsis, mitogen activated protein kinase (MAPK) pathway is of crucial importance (Strassheim et al., 2002; Abraham, 2005). The inhibition of MAPK signaling pathways is believed to suppress the inflammatory response and improve survivability in sepsis (Scherle et al., 1998; Kotlyarov et al., 1999; Meng et al., 2014). By contrast, the cholinergic antiinflammatory pathway has been recognized as a primary pathway in suppressing inflammation in sepsis and in other inflammatory conditions such as inflammatory bowel disease and rheumatoid arthritis (Webster et al., 2002; Wang et al., 2021). The released acetylcholine upon vagal activation acts via neuro-immune circuits (Borovikova et al., 2000; Kanashiro et al., 2017) to attenuate macrophage activation by facilitating the Jak2-STAT3 signaling and suppress the production of proinflammatory mediators in human monocytes (de Jonge et al., 2005; Yoshikawa et al., 2006). In addition to its antiinflammatory action, the cholinergic (vagal) innervation to the heart is pivotal to cardiovascular homeostasis and regulation of arterial baroreceptor and chemoreceptor reflexes (Capilupi et al., 2020; Rajendran et al., 2024).

Adenosine is an endogenous purine nucleoside that modulates many physiological processes via activating four subtypes of adenosine receptors (ARs) namely: A1Rs, A2aRs, A2bRs and A3Rs (Borea et al., 2018). ARs are also expressed in a wide variety of tissues including almost all types of immune cells, thereby playing a pivotal role in regulating immune responses and inflammatory conditions (Pasquini et al., 2021; Zhang et al., 2022). The activation of A1 and A3 ARs in peripheral tissues reduce the sepsis-related inflammation and mortality, and kidney and liver damage (Gallos et al., 2005; Lee et al., 2006). Nevertheless, other studies showed that elimination of A1 and A3 ARs protect against sepsis induced kidney and lung injury, respectively (Inoue et al., 2008; Wilson et al., 2014). Unlike peripheral ARs, studies on the role of central ARs in sepsis are limited and contradictory. We recently reported that central A1ARs counteract neuroinflammation and associated cardiovascular dysfunction in sepsis (El-Naggar et al., 2023) while Guo et al. (2023) reported that astrocytic A1ARs exacerbate neuroinflammation contributing to sepsis induced encephalopathy. To the best of our knowledge, no data is available on the role of central A3ARs in sepsis.

Therefore, the current study employed pharmacologic and molecular means to test the hypotheses (i) central A3ARs arbitrate cardiovascular and neuroinflammatory insults of sepsis and (ii) the cholinergic antiinflammatory pathway contributes to the A3AR/sepsis interaction. Studies were undertaken in conscious rats pre-instrumented with indwelling femoral and intracisternal catheters to assess the effect of separate or combined treatment with nicotinic and A3AR ligands on cardiovascular, autonomic, and inflammatory responses elicited by sepsis. The role of the central PI3K/MAPKs/TNFα cascade in the A3AR/nicotine interaction was also evaluated.

## 2 Materials and methods

### 2.1 Animals

Adult male Wistar rats (220–250 g) were obtained from the Animal facility of the Faculty of Pharmacy, Alexandria University, Egypt, and were maintained under controlled laboratory conditions and allowed free access to standard rat chow and tap water. All experimental protocols were approved by the Institutional Animal Care and Use Committee, Alexandria University, Egypt (Approval No. AU/06.2020.6.7.2.73) and carried out in accordance with the Declaration of Helsinki and the Guide for the Care and Use of Laboratory Animals.

### 2.2 Drugs

Betadine^®^ (povidone iodine solution 10%), heparin^®^ (heparin sodium, 5,000 I.U/mL), pencitard^®^ (1,200,000 I.U benzathine benzyl penicillin), thiopental^®^ (thiopental sodium, 500 mg vial), remicade^®^ (infliximab, 100 mg vial, Janssen Biotech, Inc.), nicotine (Merck Schuchardt OHG, Hohenbrunn, Germany), IB-MECA (N(6)-(3-iodobenzyl)-5′-N-methylcarboxamidoadenosine), PD98059 (2-(2-Amino-3-methoxyphenyl)-4H-1-benzopyran-4-one), SP600125 (1,9-Pyrazoloanthrone), VUF5574 (N-(2-Methoxyphenyl)-N′-[2-(3-pyrindinyl)-4-quinazolinyl]-urea), wortmannin (Sigma Chemical Co., St. Louis, MO, United States).

### 2.3 Induction of sepsis by cecal ligation and puncture (CLP)

Cecal ligation and puncture (CLP) was conducted as previously described (Toscano et al., 2011; El-Naggar et al., 2023) 24 h before conducting cardiovascular monitoring. The abdominal area of thiopental-anesthetized rats (50 mg/kg, i.p.) was shaved and disinfected using betadine solution. A midline laparotomy (∼1.5 cm) was performed, the cecum was exposed and one-third of the distal end, away from the ileocecal valve, was tightly ligated. The cecum was then punctured three times on the same side using a 21-gauge needle and gently compressed to extrude a small amount of fecal content into the peritoneal cavity. At the end, the cecum was returned to the abdominal cavity and the skin and underlying abdominal musculature were stitched.

### 2.4 Intracisternal cannulation

Five days before the day of experiment (i.e., 4 days before intravascular cannulation and CLP), a stainless steel guide cannula (23 G, Miami, FL, United States) was implanted into the cisterna magna of thiopental-anesthetized rats (50 mg/kg, i.p.) as previously described in our studies (El-Mas et al., 2009; El-Mas et al., 2012). The guide cannula was passed between the occipital bone and the cerebellum so that its tip protruded into the cisterna magna. The cannula was secured in place with dental luting cement (Glass Ionomer, Hangzhou, China). Each rat received an i.m. injection of benzathine benzyl penicillin (60,000 U) and was housed individually.

### 2.5 Intravascular cannulation

Intravascular cannulation was performed on the same day of CLP as previously described (El-Mas and Abdel-Rahman, 1995; El-Mas and Abdel-Rahman, 1997; El-Mas and Abdel-Rahman, 1999; El-Mas et al., 1997). Briefly, rats were anesthetized with thiopental (50 mg/kg, i.p.) and polyethylene catheters were inserted into the abdominal aorta and vena cava via the femoral artery and vein for hemodynamic measurement and i.v. drug administration, respectively. Catheters were tunneled subcutaneously, exteriorized at the back of the neck between the scapulae, flushed with heparin (100 U/mL), and plugged by stainless steel pins. One day later, the arterial catheter was connected to a BP transducer (model P23XL; Astro-Med, West Warwick, RI, United States) that was attached through MLAC11 Grass adapter cable to a computerized data acquisition system with LabChart-7 pro software (Power Lab 4/35, model ML866/P; AD Instruments Pty Ltd., Castle Hill, Australia) for the measurement of blood pressure (BP), heart rate (HR) and heart rate variability (HRV) as mentioned below.

### 2.6 Time-domain analysis of HRV

Two statistically-derived parameters of HRV were measured: SDNN, the standard deviation of NN intervals (R-R interval of normal beats) and rMSSD, the square root of the mean squared differences of successive NN intervals (Stein et al., 1994; Omar and El-Mas, 2004). The NN intervals were computed from the HR signals (i.e., the reciprocal of HR in ms). SDNN is recognized as a measure of the overall activity of the autonomic control of the heart and correlates with the total power which is the variance of NN intervals (Stein et al., 1994). rMSSD can be taken as a measure of the parasympathetic activity and correlates with the high frequency (HF) power of the spectrum (Stein et al., 1994; Berntson et al., 1997). Time-domain parameters of HRV, SDNN and rMSSD, were measured before (baseline) and at 10 min intervals after drug treatments.

### 2.7 Frequency-domain analysis of HRV

Spectral analysis of HRV was used to reflect changes in sympathetic and parasympathetic control of the heart. Frequency-domain parameters of HRV were analyzed based on the fast Fourier transform algorithm (FFT) which has the advantage of simplicity and high processing speed (Stein et al., 1994; El-Naggar et al., 2018). Spectra were integrated into two frequency bands, LF (0.25–0.75 Hz) and HF (0.75–3 Hz) bands and expressed in normalized units (LFnu and HFnu) which minimizes the effect of total power on the values of LF and HF components and reduces the effect of noise (Sztajzel, 2004). The LF/HF ratio is taken as a measure of the cardiac sympathovagal balance. Frequency-domain parameters of HRV were estimated before (baseline) and at 10 min intervals after drug treatments.

### 2.8 Immunohistochemistry

Immunohistochemical analysis was performed according to the technique described in previous studies (Chen and Sun, 2006; Helmy et al., 2015). The protein expressions of NFκB and NOX2 were determined in brainstem areas of rostral ventrolateral medulla (RVLM). Rat brainstems were fixed in 10% formalin and embedded in paraffin blocks. Approximately 5 μm sections of rat brainstem (−12.0 mm relative to bregma) (El-Mas and Abdel-Rahman, 1995; El-Mas and Abdel-Rahman, 1997) were cut and placed on positively charged adhesion glass slides (Epredia™, Braunschweig, Germany), then deparaffinized in xylene and rehydrated in a series of descending ethanol concentrations (100%, 95% and 70%). Heat-induced epitope retrieval was performed by immersing the slides in coplin jars containing 10 mM citrate buffer solution and incubated in a microwave at power 100 for 1 min then power 30 for 9 min. Endogenous peroxidases were blocked by 3% hydrogen peroxide for 10 min. The diluted primary polyclonal antibodies (1:300, rabbit anti-NFκB p65, Bioss ™, United States) and (1:250, rabbit anti-NOX2, ThermoFisher, United States) were applied to the slides and then sections were incubated at 4 C overnight. The secondary antibody (HRP conjugate) was applied for 30 min. The chromogen 3,3′-diaminobenzidine (DAB) was prepared and applied as instructed by the manufacturer for protein visualization. Slides were counterstained with hematoxylin and dipped in ascending concentrations of alcohol and then xylene. Images were taken by OptikamB9 digital camera (Optika ^®^ microscopes, Italy) and Fiji Image J software version 1.51n (National Institutes of Health, Bethesda, MA, United States) was employed to measure the area fraction of DAB positive staining in brainstem areas of RVLM.

### 2.9 Protocols and experimental design

#### 2.9.1 Role of central A3ARs in nicotinic modulation of septic manifestations

This experiment investigated the effects of nicotine on cardiovascular and autonomic derangements observed in septic rats and its possible modulation by central A3ARs. A total of seven groups of conscious rats (n = 8 each) were employed and designed to receive one of the following regimens: (i) sham + saline (i.v.), (ii) CLP + saline (i.v.), (iii) CLP + nicotine (100 μg/kg, i.v.) (Sallam et al., 2018), (iv) CLP + IB-MECA (selective A3AR agonist, 4 µg/rat, i.c.) (Chen et al., 2006), (v) CLP + IB-MECA (i.c.) + nicotine (100 μg/kg, i.v.), (vi) CLP + VUF5574 (A3AR antagonist, 2 µg/rat, i.c.) (El-Mas et al., 2011), or (vii) CLP + VUF5574 (i.c.) + nicotine (100 μg/kg, i.v.). A 10-min interval was allowed between the two successive treatments of each regimen, and hemodynamic monitoring continued for 2 h after the last treatment. Notably, the high potency and selectivity of IB-MECA and VUF5574 as A3AR agonist and antagonist, respectively, have been established. VUF5574 has a K(i) value of 4 nM and is at least 2,500-fold selective for A3AR over A1AR and A2ARs (van Muijlwijk-Koezen et al., 2000). On the other hand, IB-MECA has a K(i) value of 1.1 nM at A3ARs which is 50-fold higher than K(i) for A1 and A2a ARs (Gallo-Rodriguez et al., 1994).

**FIGURE 1:**
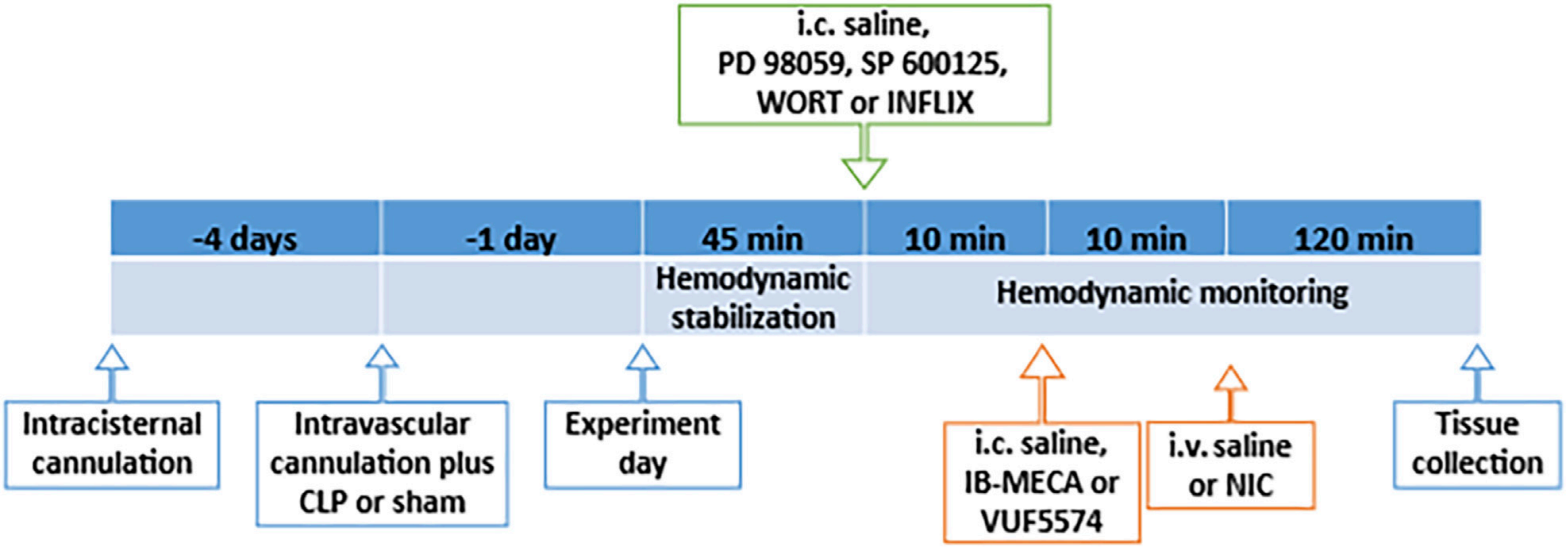
Diagrammatic representation of the timeline of surgical procedures and drug regimens.

Changes in MAP, HR and HRV parameters were computed at 10 min intervals. At the end of the observation period, rats were euthanized with an overdose of thiopental (100 mg/kg), brainstems were quickly removed, fixed in 10% formaldehyde solution, and processed for immunohistochemical measurement of the protein expression of NFκB and NOX2 as described above. The diagrammatic representation of the timeline of surgical procedures and drug regimens employed in the experiments is depicted in Figure 1.

#### 2.9.2 Role of PI3K/MAPK/TNFα signaling on IB-MECA-nicotine septic interaction

Pharmacologic studies were utilized to evaluate the role of the PI3K/MAPK/TNF-α cascade in the counteraction by IB-MECA of cholinergic defenses against sepsis. Additional four groups of conscious rats (n = 8 each) subjected to CLP operation were allocated to receive one of the following drug regimens: (i) PD98059 (MAPK-ERK inhibitor, 10 µg/rat, i.c.) (Sallam et al., 2016; Sallam et al., 2017) + IB-MECA (4 µg/rat, i.c.) + nicotine (100 μg/kg, i.v.), (ii) SP600125 (MAPK-JNK inhibitor, 30 µg/rat, i.c.) (Sallam et al., 2016; Sallam et al., 2017) + IB-MECA (4 µg/rat, i.c.) + nicotine (100 μg/kg i.v.) (iii) wortmannin (PI3K inhibitor, 0.5 µg/rat, i.c.) (Sallam et al., 2016; Sallam et al., 2017) + IB-MECA (4 µg/rat, i.c.) + nicotine (100 μg/kg, i.v.) or (iv) infliximab (TNF-α inhibitor, 100 µg/rat, i.c.) (Dadsetan et al., 2016; Mohamed et al., 2023) + IB-MECA (4 µg/rat, i.c.) + nicotine (100 μg/kg, i.c.). A 10-min interval was allowed between each two successive treatments, and hemodynamic monitoring continued for 2 h after the last treatment. Changes in MAP, HR and HRV parameters were computed at 10 min intervals. At the end of hemodynamic monitoring, rats were euthanized with an overdose of thiopental (100 mg/kg), brainstems were quickly removed, fixed in 10% formaldehyde solution, and processed for immunohistochemical measurement of the protein expression of NFκB and NOX2 as described earlier. The diagrammatic representation of the timeline of surgical procedures and drug regimens employed in the experiments is depicted in Figure 1.

### 2.10 Statistical analysis

Values are expressed as means ± standard error of the mean (SEM). The area under the curve (AUC) was calculated for each parameter to express the cumulative effect of tested regimens over the entire period of hemodynamic monitoring. AUCs were computed by GraphPad Prism 8.0.2. using trapezoidal integration and zero line as the baseline. Peaks that go below the baseline were also considered by computing the “net area” which represents the area of peaks below the baseline minus the area of peaks above the baseline. The unpaired Student’s *t* test was used to test for significance between two independent groups. The one-way or repeated measures ANOVA followed by the Tukey’s *post hoc* test was used to test for significance among multiple groups. These analyses were performed by GraphPad InStat, software release 3.05. Probability levels less than 0.05 were considered significant.

## 3 Results

### 3.1 Cardiovascular and autonomic effects of sepsis

**FIGURE 2:**
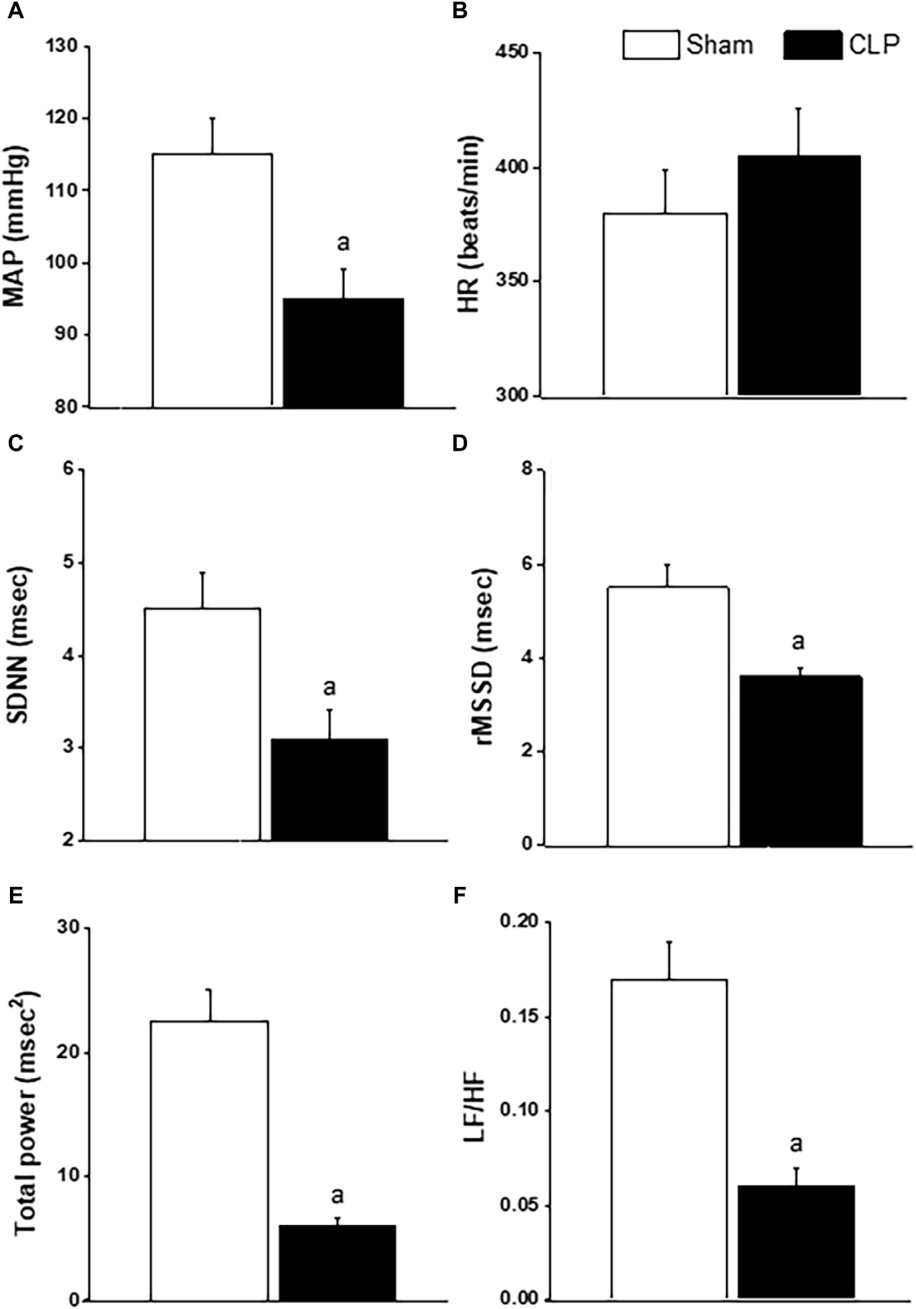
Effect of sepsis on blood pressure (BP, panel **(A)**, heart rate (HR, panel **(B)**, time-domain parameters of HRV (SDNN, panel **(C)**; rMSSD, panel **(D)**] and frequency-domain parameters of HRV [total power, panel **(E)**; LF/HF, panel **(F)**] in male rats. ^a^ *p* < 0.05 vs. sham values.

Baseline values of BP, HR and HRV measured 24 h after CLP or sham operation are depicted in Figure 2. CLP significantly reduced in BP (Figure 2A) while having no effect on HR (Figure 2B). CLP also produced significant reductions in the two time-domain indices of HRV, the standard deviation of NN intervals (SDNN, Figure 2C) and root mean squared differences of NN intervals (rMSSD, Figure 2D). The total power band of the HRV spectrum (Figure 2E) as well as the LF/HF ratio (Figure 2F) were significantly less in CLP rats than in respective values of sham rats, denoting a shift in cardiac sympathovagal balance towards parasympathetic predominance.

### 3.2 Central A3ARs offset the favorable cardiovascular effects of nicotine against sepsis

**FIGURE 3:**
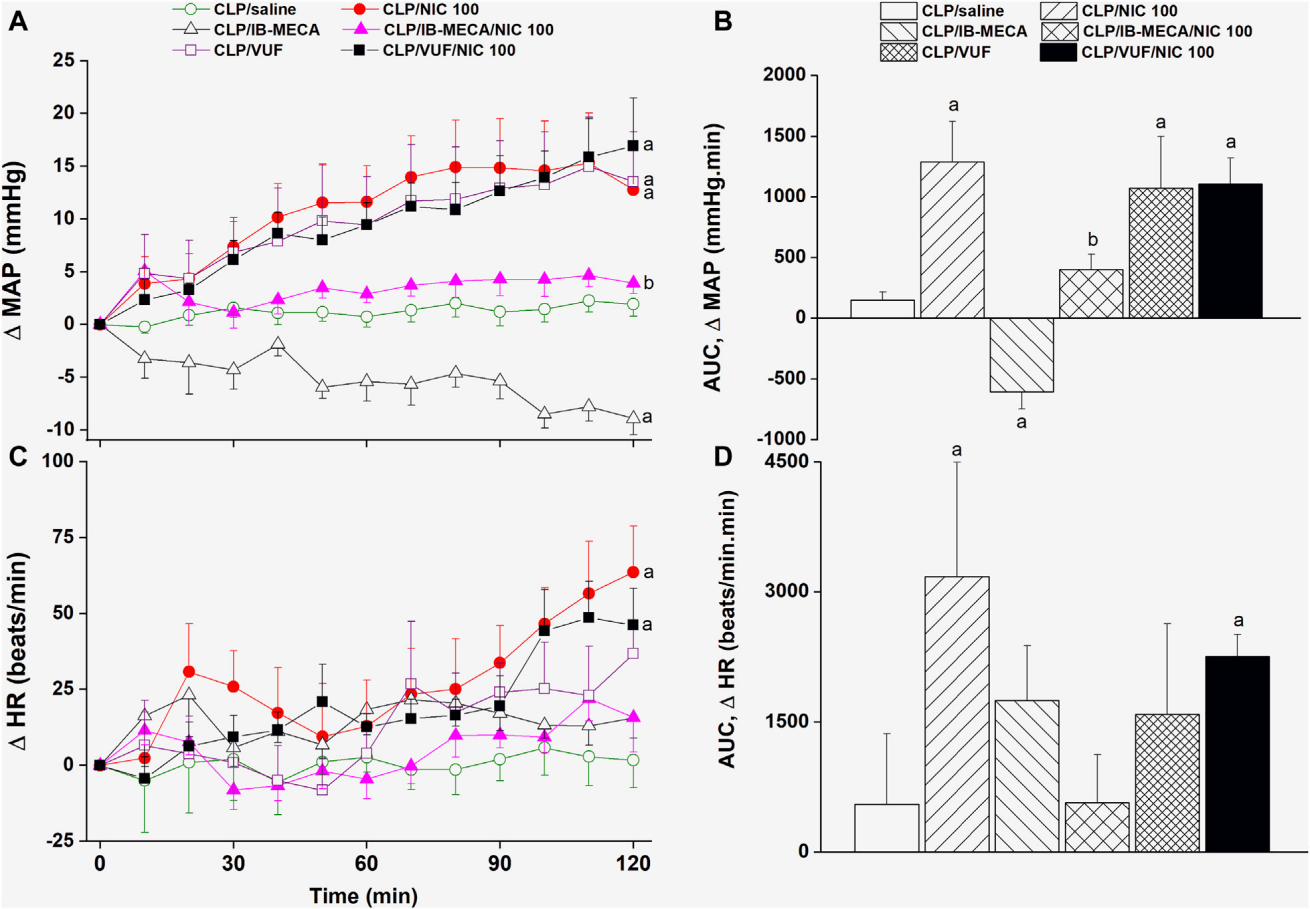
Effects of central adenosine A3 receptor activation (IB-MECA) or blockade (VUF5574) on the nicotine-evoked changes in time-course [panels **(A–C)**] and cumulative values [AUCs, panels **(B–D)**] of MAP and HR in septic male rats. ^a^ *p* < 0.05 vs. “CLP/saline”, ^b^ *p* < 0.05 vs. “CLP/NIC”.

**FIGURE 4:**
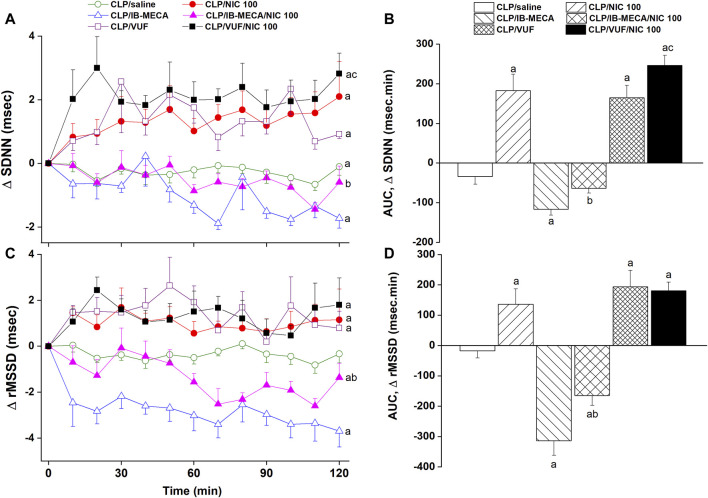
Effects of central adenosine A3 receptor activation (IB-MECA) or blockade (VUF5574) on the nicotine-evoked changes in time-course [panels **(A–C)]** and cumulative values [AUCs, panels **(B–D)**] of time-domain indices of HRV (SDNN, rMSSD) in septic male rats. ^a^ *p* < 0.05 vs. “CLP/saline”, ^b^ *p* < 0.05 vs. “CLP/NIC”, ^c^ *p* < 0.05 vs. “CLP/VUF”.

**FIGURE 5:**
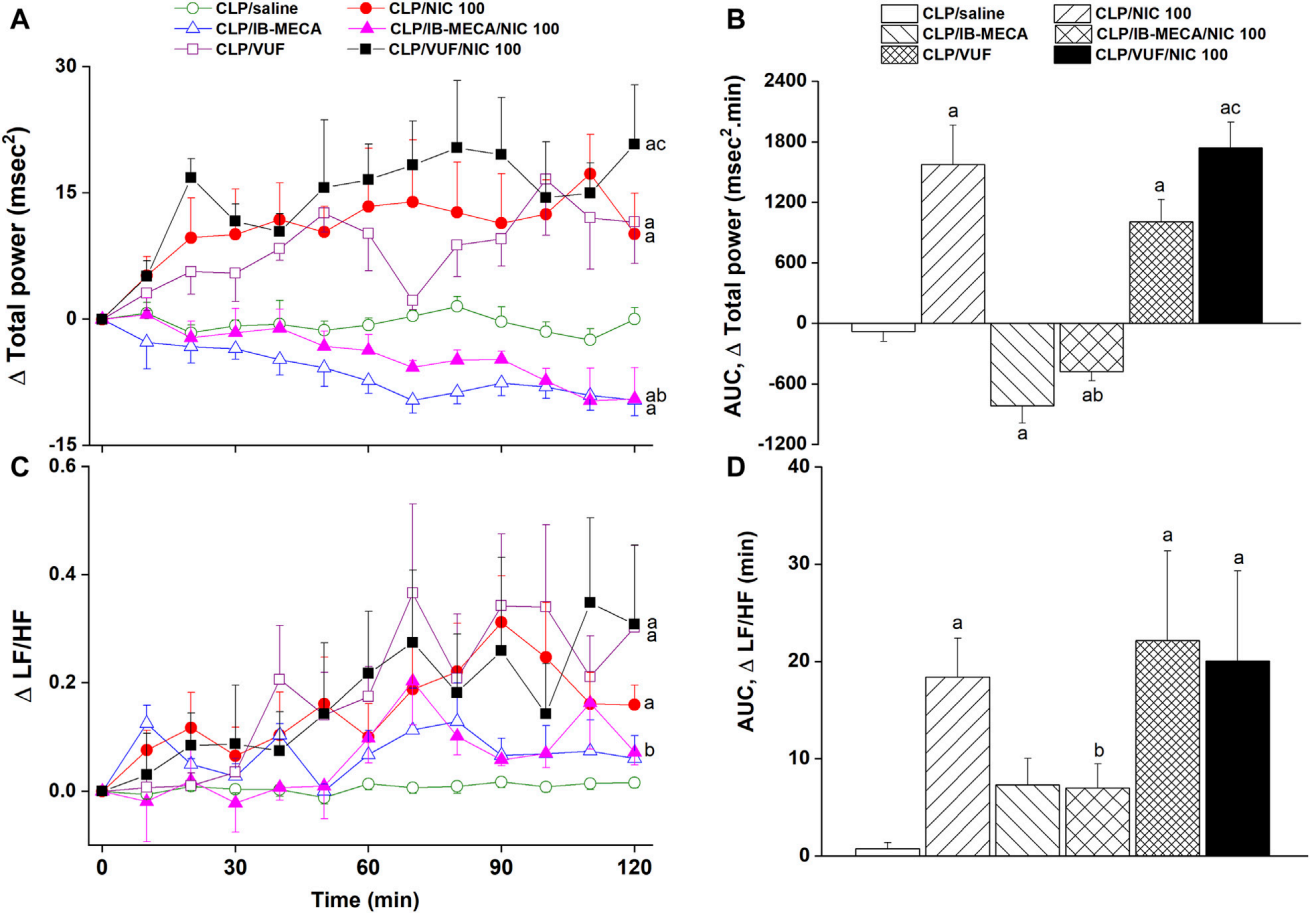
Effects of central adenosine A3 receptor activation (IB-MECA) or blockade (VUF5574) on the nicotine-evoked changes in time-course [panels **(A–C)**] and cumulative values [AUCs, panels **(B–D)**] of frequency-domain indices of HRV (total power, LF/HF ratio) in septic male rats. ^a^ *p* < 0.05 vs. “CLP/saline”, ^b^ *p* < 0.05 vs. “CLP/NIC”, ^c^ *p* < 0.05 vs. “CLP/VUF”.

The effects of pharmacologic manipulation of central A3ARs on septic responses and their interaction with nicotine are depicted in Figures 3–5. Intravenous administration of nicotine (100 μg/kg) caused significant increases in MAP (Figures 3A, B) and HR (Figures 3C, D) compared with respective values in saline-treated CLP rats, confirming the ability of nicotine to reverse the hypotensive response initially caused by the septic challenge and shown in Figure 2A. Likewise, HRV analysis revealed that the sepsis-evoked reductions in time (SDNN and rMSSD, Figure 4) and frequency (total power, Figures 5A, B) domain indices of HRV were significantly upturned by nicotine. Nicotine also enhanced the depressed sympathovagal balance in CLP rats as indexed by the rise in the LF/HF ratio (Figures 5C, D). Together, Figures 4, 5 demonstrate the ability of nicotine to alleviate hemodynamic and HRV disturbances induced by sepsis.

The i.c. treatment of CLP rats with the selective A3AR agonist IB-MECA (4 µg/rat) accentuated the cardiovascular and HRV responses elicited by sepsis. In other words, IB-MECA significantly reduced MAP (Figures 3A, B), overall HRV indices (SDNN, Figures 4A, B; total power, Figures 5A, B) and parasympathetic cardiotonic activity (rMSSD, Figures 4C, D). Further, IB-MECA blunted the pressor, tachycardic, and rises in HRV indices produced by subsequent administration of nicotine (Figures 3–5).

Alternatively, the blockade of central A3ARs by i.c. VUF5574 (2 µg/rat) in CLP rats produced effects that mimicked those produced by nicotine. VUF5574 significantly increased MAP (Figures 3A, B), and time (Figure 4) and frequency (Figure 5) indices of HRV indices. The cardiovascular and HRV changes evoked by the combined VUF5574/nicotine regimen were similar caused by nicotine alone (Figures 3–5).

### 3.3 Central PI3K/MAPKs/TNFα cascade modulates the IB-MECA/nicotine interaction

**FIGURE 6:**
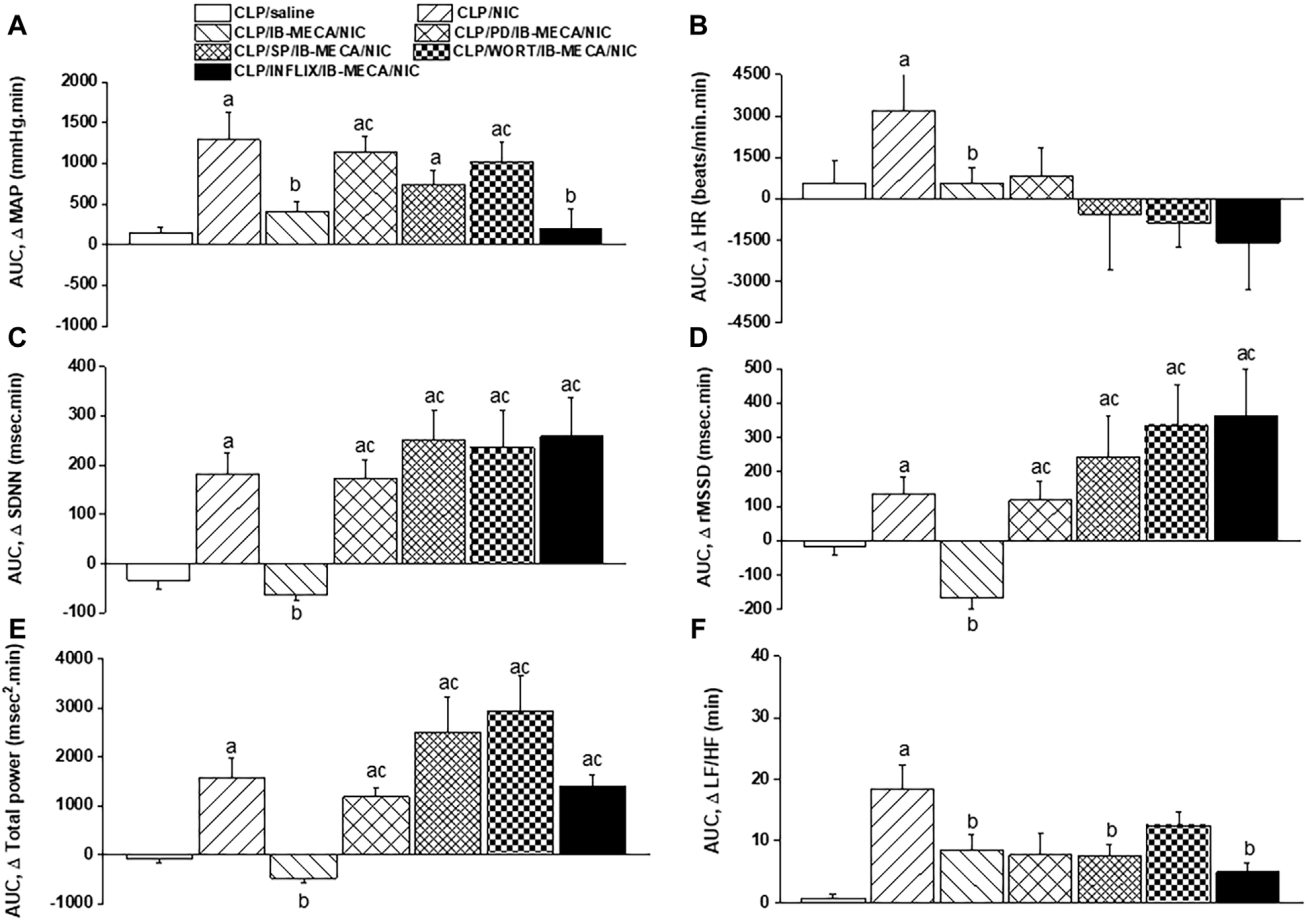
Effects of intracisternal administration of PD98059 (MAPK-ERK inhibitor), SP600125 (MAPK-JNK inhibitor), wortmannin (PI3K inhibitor) or infliximab (TNFα inhibitor) on IB-MECA modulation of nicotine-induced cumulative changes in MAP, HR [panels **(A,B)**], time-domain indices of HRV [SDNN, rMSSD; panels **(C,D)**] and frequency-domain indices of HRV [total power, LF/HF ratio; panels **(E,F)**] in septic (CLP) male rats. ^a^ *p* < 0.05 vs. “CLP/saline”, ^b^ *p* < 0.05 vs. “CLP/NIC”, ^c^ *p* < 0.05 vs. “CLP/IB-MECA/NIC”.

The influence of pharmacologic inhibition of individual components of the PI3K/MAPKs/TNFα pathway on the IB-MECA-nicotine interaction in septic rats was evaluated. Figures 6A, B demonstrate that the depressant action of IB-MECA on nicotine hypertension was eliminated following central inhibition of MAPK-ERK, MAPK-JNK, or PI3K evoked by i.c. administration of PD98059 (10 µg/rat), SP600125 (30 µg/rat), and wortmannin (0.5 µg/rat), respectively. By contrast, the counteraction of nicotine hypertension by IB-MECA was preserved in rats treated with i.c. infliximab (TNFα inhibitor, 100 µg/rat). Analysis of HRV illustrated that i.c. treatment with each of the above inhibitors (PD98059, SP600125, wortmannin or infliximab) reversed the inhibitory effects of IB-MECA on the nicotine-induced increments in time-domain indices of total cardiac autonomic control (SDNN, Figure 6C) as well as on cardiac vagal cardiotonic activity (rMSSD, Figure 6D). The spectral index of total autonomic activity (total power, Figure 6E) was also reversed by the tested inhibitors, whereas the falls caused by IB-MECA in the cardiac sympathovagal balance (LF/HF ratio, Figure 6F) remained unaltered by any of these inhibitors.

### 3.4 Brainstem expression of NFκB and NOX2

**FIGURE 7:**
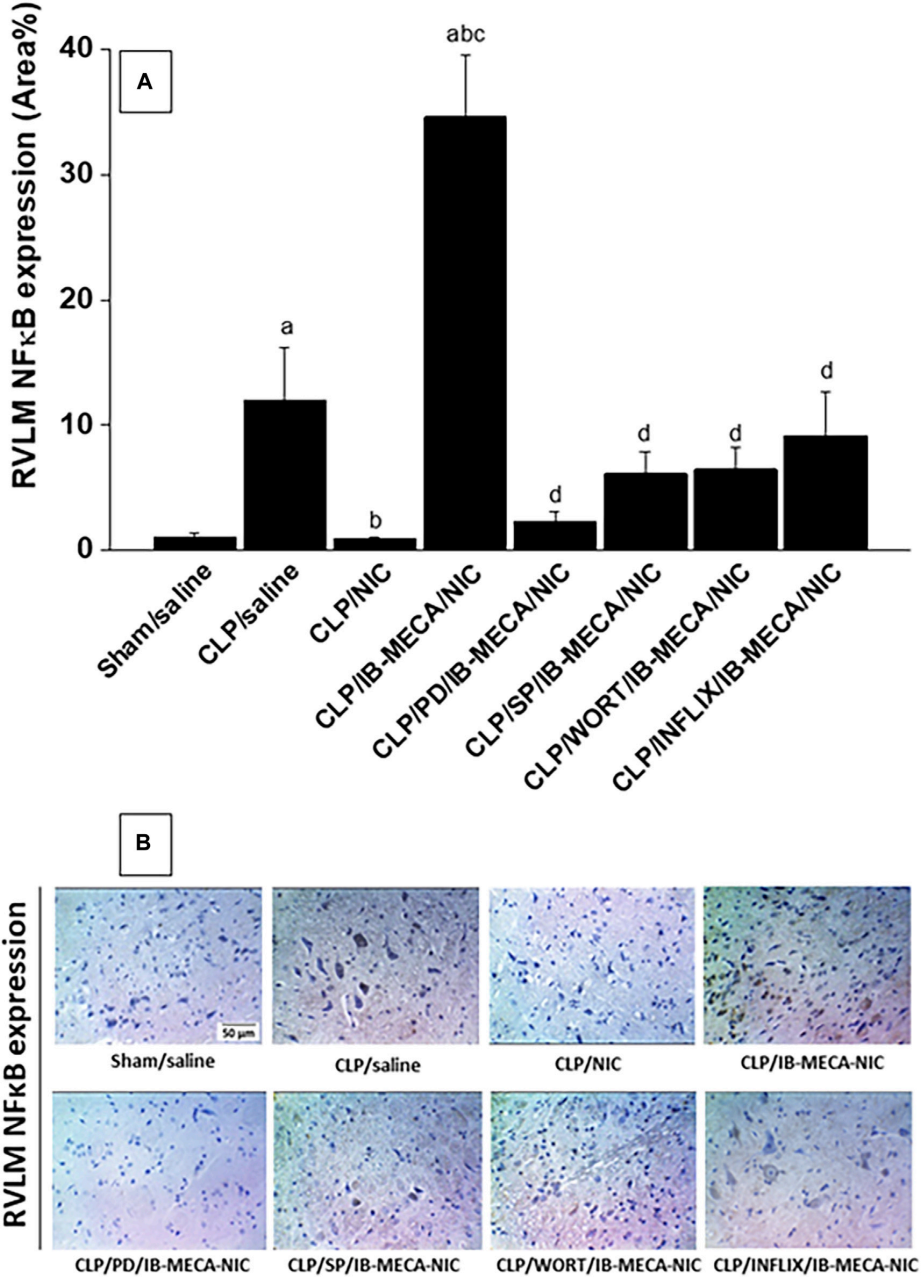
Panel **(A)** shows the effects of central adenosine A3 receptor activation (IB-MECA) on the nicotine-evoked downregulation of elevated NFκB expression in the RVLM of septic rats in absence and presence of PD98059 (MAPK-ERK inhibitor), SP600125 (MAPK-JNK inhibitor), wortmannin (PI3K inhibitor) or infliximab (TNFα inhibitor). Representative images for immunostained sections are shown in panel **(B)**. ^a^ *p* < 0.05 vs. “sham/saline”, ^b^ *p* < 0.05 vs. “CLP/saline”, ^c^ *p* < 0.05 vs. “CLP/NIC”. ^d^ *p* <0.05 vs. “CLP/IB-MECA/NIC”.

**FIGURE 8:**
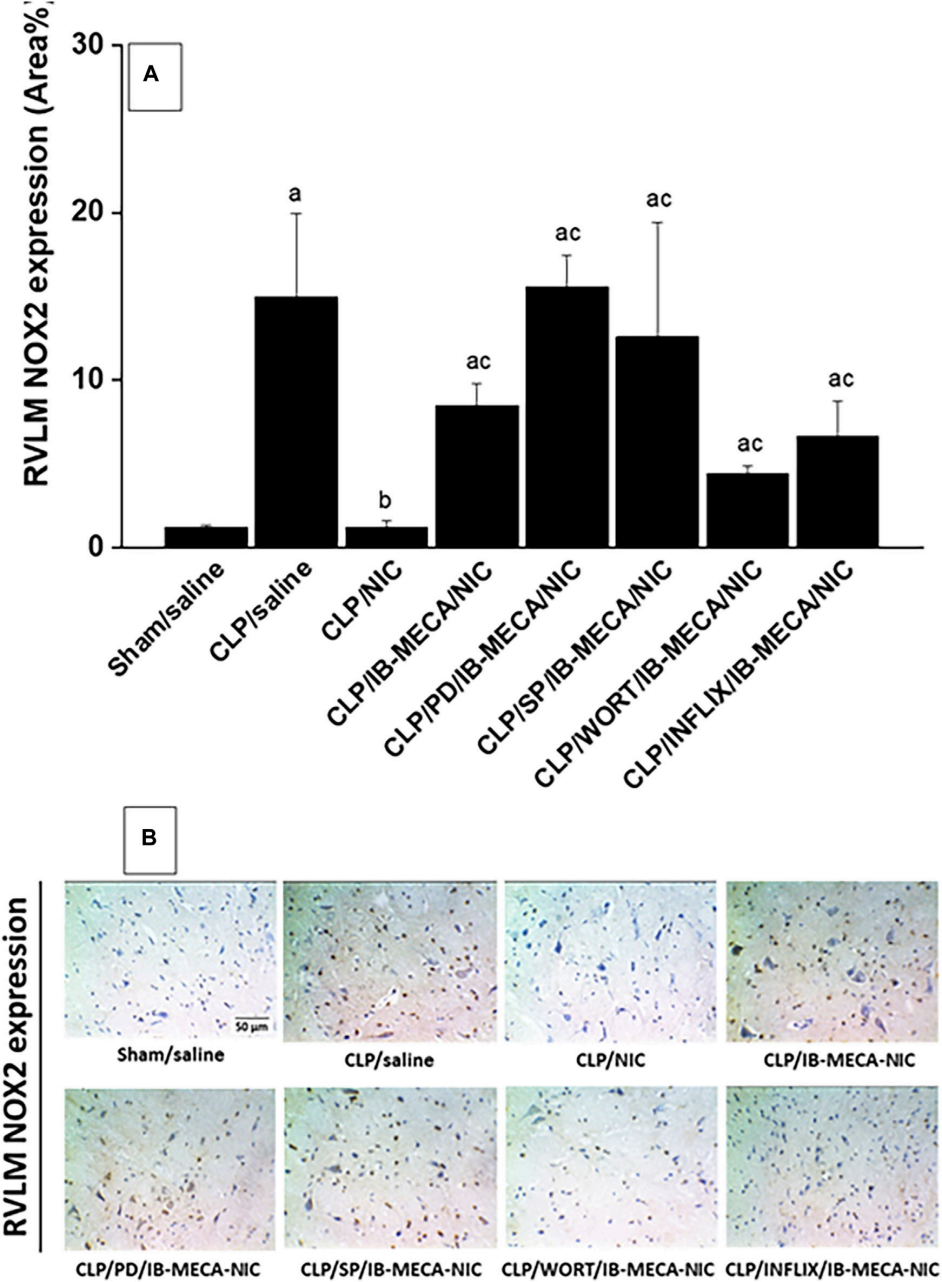
Panel **(A)** shows the effects of central adenosine A3 receptor activation (IB-MECA) on the nicotine-evoked downregulation of elevated NOX2 expression in the RVLM of septic rats in absence and presence of PD98059 (MAPK-ERK inhibitor), SP600125 (MAPK-JNK inhibitor), wortmannin (PI3K inhibitor) or infliximab (TNFα inhibitor). Representative images for immunostained sections are shown in panel **(B)**. ^a^ *p* < 0.05 vs. “sham/saline”, ^b^ *p* < 0.05 vs. “CLP/saline”, ^c^ *p* < 0.05 vs. “CLP/NIC”.

Immunohistochemical analysis showed that the protein expression of the proinflammatory NFκB (Figure 7A) and oxidant NOX2 (Figure 8A) in neuroanatomical areas of the brainstem RVLM were significantly increased in CLP compared with sham rats. The CLP-associated overexpressed signals of NFκB and NOX2 were restored back to near-sham levels after treatment of CLP rats with nicotine and resurfaced upon simultaneous administration of the A3AR agonist IB-MECA (Figures 7, 8). Consistent with the abovementioned cardiovascular data, i.c. administration of PD98059, SP600125, wortmannin, or infliximab opposed the counteracting effects of IB-MECA on nicotine downregulation of NFκB neuronal expression (Figure 7A). On the contrary, none of these inhibitors alleviated the inhibitory action of IB-MECA on nicotine-evoked downregulation of NOX2 expression (Figure 8A). Representative images of the immunostained neuronal areas are shown in Figures 7B, 8B.

## 4 Discussion

The involvement of circulating as well as cardiac and renal proinflammatory cytokines in the cholinergic modulation of cardiovascular sequels of sepsis has been repeatedly investigated in previous reports from our laboratory (Sallam et al., 2018; Wedn et al., 2020; El-Naggar et al., 2023). This study is the first to report on the role of central pathways of A3ARs in the cholinergic modulation of cardiovascular and inflammatory responses to sepsis. The data revealed that nicotine reversed the hypotensive response elicited by sepsis and simultaneous reduction in HRV and upregulation of brainstem NFκB and NOX2 expression. Selective activation of A3AR by IB-MECA aggravated the already existing hypotension and cardiac autonomic dysfunction and counterbalanced the favorable cardiovascular and brainstem neuroinflammatory actions evoked by nicotine. The hostile response to A3AR agonism was mostly ameliorated after central pharmacological inhibition of PI3K, MAPK-ERK, MAPK-JNK, or TNFα. Further, these inhibitors restored the nicotine downregulation of heightened RVLM NFκB, but not NOX2, expression. Collectively, the data implicate central PI3K/MAPK/TNFα signaling in the A3AR counteraction of the ameliorative action of nicotine on cardiovascular and neuroinflammatory derangements in sepsis.

Myocardial dysfunction and altered hemodynamics are major complications of sepsis and septic shock (Hochstadt et al., 2011; Greer, 2015). More specifically, HRV features have been recognized as key predictors of organ dysfunction in sepsis. For example, human electrocardiographic recordings showed that 3-h measurements of time and frequency indices of HRV is predictive of progressive organ dysfunction during early days of sepsis (van Wijk et al., 2023). Others reported remarkable negative correlations between HRV-LF power and circulating IL-6 in hospitalized septic patients on the one hand, and the decline in blood pressure on the other hand (Tateishi et al., 2007). Lear et al. (2014) reported that the inflammatory response to acute endotoxemia in sheep is associated with a drop in blood pressure together with consecutive increases and decreases in HRV. In the current study, we showed that CLP, the gold standard model of sepsis (Dejager et al., 2011), resulted in hypotension and suppression of time- and frequency-domain indices of HRV. This gains support from the observations that CLP rats exhibited (i) diminished overall cardiac autonomic control as indexed by the falls in SDNN and total power, and (ii) shifts in sympathovagal balance towards parasympathetic predominance as verified by the reductions in LF/HF ratios. The increased vagal activity and depressed sympathetic modulation of the heart have been reported in septic patients (Hsu et al., 2020). Clinically, the depressed HRV in septic patients correlates with worsened prognosis and mortality in sepsis (Barnaby et al., 2002; Pontet et al., 2003; de Castilho et al., 2018). Further, the counteraction by systemically administered nicotine of the hypotensive and cardiac autonomic neuropathic actions of sepsis reinforces the shielding action of the cholinergic antiinflammatory pathway against sepsis (Sallam et al., 2018; Sallam et al., 2019; El-Naggar et al., 2023).

Although the immunomodulatory function of adenosine in peripheral tissues in sepsis pathophysiology has been recognized (Zhang et al., 2022), reports on the role of central adenosinergic pathways in sepsis are scarce. For instance, we recently demonstrated that myocardial and brainstem A1ARs act tonically to boost the protection conferred by the cholinergic antiinflammatory system against cardiomyopathic sequels of sepsis (El-Naggar et al., 2023). Similarly, Igarashi et al. (2023) demonstrated a favorable role for central A2BARs in the vagally-dependent diminution of mortality risk in endotoxemia. Contrary to such apparently privileged roles of A1ARs and A2BARs in the fight against sepsis, the current data suggests an exacerbating action for central A3ARs on cardiovascular irregularities induced by sepsis. Such distinct effect of A3ARs is supported by experiments that tested the effect of the A3AR agonist IB-MECA alone or combined with nicotine. Indeed, the hypotension and cardiac autonomic dysfunction induced by sepsis were significantly intensified following central administration of IB-MECA into the cisterna magnum. Further, IB-MECA abolished the rises in BP and time and spectral indices of HRV indices evoked by consequent treatment with nicotine in septic rats. These findings highlight a pivotal role for downregulation of the cholinergic antiinflammatory pathway in the worsened cardiovascular profile in septic rats upon A3AR activation.

To further consolidate the role of A3ARs in sepsis pathophysiology, we tested the influence of central A3AR blockade by VUF5574 on septic responses. Fascinatingly, we found that VUF5574 acted in a similar fashion to nicotine, i.e., caused remarkable elevations in BP and time- and frequency-domain HRV parameters. Likewise, the use of VUF5574 or nicotine significantly increased the cardiac sympathovagal balance (LF/HF ratio, Figure 5D), inferring a rise in cardiac sympathetic activity. The separate effects of nicotine and VUF5574 in this context were indistinguishable, both qualitatively and quantitatively, suggesting positive roles for the enhanced HRV and sympathetic activity of the heart, and probably other sympathetic neural beds, in the counterbalancing actions of the two drugs against cardiovascular manifestations of sepsis. These observations together with the data of the agonistic IB-MECA study point clearly toward a tonic facilitatory role for central A3ARs in mediating cardiovascular and autonomic aberrations induced by sepsis. A similar role for A3ARs has been described by Inoue et al. (2008) who reported less lung injury and improved survivability in A3AR-knockout CLP mice compared with wild-type septic mice.

The current study investigated the role of central inflammatory and oxidative molecules in the A3AR-nicotine interaction. This was accomplished by immunohistochemical determination of NFκB and NOX2 expression in neuroanatomical areas of the RVLM. NFκB is a pivotal transcription factor that predicts sepsis severity and mortality and induces a variety of downstream proinflammatory effectors (Barnes and Karin, 1997; Böhrer et al., 1997; Arnalich et al., 2000; Abraham, 2003; Basak and Akashi-Takamura, 2024). NOX2 is a superoxide generating enzyme that positively relates to NFκB signaling and to the oxidative burst featured during sepsis (Ouyang et al., 2024). Consistent with these reports, our data revealed about tenfold increase in the expression of NFκB and NOX2 in the RVLM of CLP, compared with sham, rats. More importantly, we also found that these inflammatory and oxidative upsets disappeared upon systemic exposure of Septic rats to nicotine and reinstated after simultaneous i. c. administration of the A3AR agonist IB-MECA. Together, these novel findings suggest a prime role for the inflammatory and oxidative response incited by central A3AR activation in the depression of the cholinergic defense against cardiovascular dysfunction and autonomic neuropathy induced by sepsis. Notably, immunohistochemical studies were specifically performed in the RVLM because this ventrolateral medullary area of the brainstem plays a key role in cardiovascular homeostasis (Saha, 2005) and in the central processing of peripheral inflammatory signals of sepsis (Sallam et al., 2019; Abuiessa et al., 2020). Peripheral inflammatory signals are believed to enter the brain through the brainstem nucleus of the solitary tract, which acts through its polysynaptic neuronal projections to the RVLM and other medullary and hypothalamic nuclei to integrate cardiovascular and inflammatory irregularities of endotoxemia (Lin et al., 1999; Pavlov et al., 2003; Sirivelu et al., 2012). We have previously reported that the upregulation of brainstem neuroinflammatory pathways of NFκB mediates septic hypotension and autonomic depression (Sallam et al., 2018; Sallam et al., 2019; El-Naggar et al., 2023).

We performed more pharmacologic and molecular studies to investigate whether the PI3K/MAPK signaling provokes the restraining influence of A3ARs on the cholinergic antiinflammatory action. In spite of the pathogenic role of MAPKs in microglial neuroinflammation as well as downstream activation of NFκB and other proinflammatory mediators (Sallam et al., 2016; An et al., 2020), contradictory reports are available regarding whether A3ARs and MAPK inflammatory signaling are interrelated (Martin et al., 2006; Ye et al., 2020). Our data showed that central inhibition of PI3K (wortmannin), MAPK-ERK (PD 98059), or MAPK-JNK (SP600125) comparably counterbalanced the depressant effects of IB-MECA on the advantageous nicotine responses in septic animals. More specifically, the IB-MECA-evoked rises in the RVLM NFκB expression and associated falls in blood pressure and time and frequency indices of overall HRV were all circumvented after the administration of each of the above inhibitors. It is tempting to speculate that the presence of intact and functional PI3K/MAPK-ERK/MAPK-JNK/NFκB cascade is a pre-requisite for switching off the protection conferred by the cholinergic antiinflammatory pathway against the adverse cardiovascular effects of sepsis.

Contrary to NFκB, RVLM-NOX2 does not seem to contribute to the PI3K/MAPK-dependent IB-MECA/nicotine interaction. Indeed, our data showed that the rise caused by IB-MECA in RVLM NOX2 expression was preserved following central inhibition of PI3K, MAPK-ERK, or MAPK-JNK. A variety of NOX isoforms have been identified and shown to distinctly contribute to sepsis pathophysiology. In one study, NOX4, but not NOX1 or NOX2, is implicated in septic manifestations of acute lung injury and endothelial dysfunction (Jiang et al., 2020). Others reported that NOX1 contributes to cardiomyocyte apoptosis and ventricular systolic dysfunction (Matsuno et al., 2012) while NOX4 mediates renal tubular inflammation, apoptosis and mitochondrial dysfunction in sepsis (Li et al., 2023). NOX2 relates to cognitive impairment, neuronal hyperexcitability and seizures in sepsis (Hernandes et al., 2014; Huang et al., 2018; Huang et al., 2020). That said, more studies are warranted to investigate the possible contribution of other NOX isoforms to the A3AR/cholinergic interaction in septic cardiovascular aberrations.

TNFα has long been considered as a primary offensive molecule in sepsis severity and mortality (Damas et al., 1989; Debets et al., 1989; Marks et al., 1990). Notwithstanding, our results revealed inconsistent effects for central inhibition of TNFα by infliximab. Whereas infliximab did effectively reverse the IB-MECA-induced increments in RVLM-NFκB expression and decrements in total HRV markers (SDNN and total power) and cardiac vagal activity (rMSSD), it failed to alter the associated falls in blood pressure and cardiac sympathovagal balance (LF/HF ratio). The disparity in the way TNFα inhibition interfered with the neuroinflammatory and cardiovascular autonomic effects of IB-MECA suggests the involvement of both TNFα-dependent and -independent inflammatory pathways in the A3AR-cholinergic interaction. While TNFα and IL-1 activate the canonical NFκB pathway, other TNF family of cytokines such as CD40 and lymphotoxin beta can activate the non-canonical pathway (Sun, 2011; Hayden and Ghosh, 2014; Lu et al., 2021). More studies are needed to investigate this possibility.

It is imperative to comment on the role of the autonomic activity in sepsis progression as well as in the cholinergic antiinflammatory response to the septic insult. Despite the current reductions in time and frequency indices of HRV, the associated decline in LF/HF ratio is indicative of a shift in the cardiac sympathovagal balance towards parasympathetic dominance. The latter has been implicated in cardiomyopathic and hypotensive actions of sepsis (Mink et al., 2007; El-Mas et al., 2011). Further, proinflammatory cytokines are believed to suppress central sympathetic vasomotor tone and cardiac baroreflex mechanisms, which might account for the loss of vascular resistance during severe bacterial infections (Sayk et al., 2008; Milanez et al., 2022). Paradoxically, a possible defensive role for the parasympathetic nervous system against the cytokine storm provoked by sepsis has also been reported (Borovikova et al., 2000). This view receives support from the observation that subdiaphragmatic vagotomy inhibits behavioral and neural effects of peripherally administered LPS, thus implicating vagal afferent in the central transmission of peripheral immune signals (Hosoi et al., 2005). Remarkably, the reversal of septic cardiovascular responses by the cholinergic stimulant nicotine or the adenosine A3R receptor blocker VUF in the current study suggests a therapeutic potential for either drug therapy in the setting of sepsis. Nevertheless, the data argue against an additional advantage for the combined nicotine/VUF therapy because its effect was not significantly different from those of respective individual treatments.

Collectively, integrative and molecular data of the current study demonstrate that central A3ARs act tonically to exacerbate cardiovascular, autonomic, and neuroinflammatory sequels of sepsis and simultaneously counterbalance the shielding effect of the cholinergic antiinflammatory pathway against these insults. The study also highlights a therapeutic potential for A3AR blockade against cardiovascular derangements of sepsis. Further experimental and clinical investigations are necessary to validate this presumption.

## Data Availability

The original contributions presented in the study are included in the article/Supplementary Material, further inquiries can be directed to the corresponding author.

## Abbreviations

AR, adenosine receptor; AUC, area under the curve; BP, blood pressure; CLP, cecal ligation and puncture; DPCPX, 1,3-dipropyl-8-cyclopentylxanthine; ERK, extracellular signal-regulated kinase; HF, high frequency; HR, heart rate; HRV, heart rate variability; i.c., intracisternal; JNK, c-Jun N-terminal Kinase; LF, low frequency; MAP, mean arterial pressure; MAPK, mitogen activated protein kinase; NFκB, nuclear factor kappa B; NOX, nicotinamide adenine dinucleotide phosphate (NADPH) oxidases; PI3K, phosphoinositide-3 kinases; rMSSD, the square root of the mean squared differences of successive N-N intervals; RVLM, rostral ventrolateral medulla; SDNN, the standard deviation of the NN intervals; TNF-α, tumor necrosis factor-α.
